# Evaluation of Pulmonary Blood Flow, Right Atrium, Right Ventricle, and Pulmonary Artery in Patients After Pneumonectomy

**DOI:** 10.3390/jcm14196793

**Published:** 2025-09-25

**Authors:** Michał Stępkowski, Małgorzata Edyta Wojtyś, Norbert Wójcik, Krzysztof Safranow, Jarosław Pieróg, Dawid Kordykiewicz, Jacek Szulc, Tadeusz Sulikowski, Konrad Jarosz, Tomasz Grodzki, Janusz Wójcik

**Affiliations:** 1 Polish-American Heart Clinics Cardiovascular Center in Kędzierzyn-Koźle, Roosevelta 2, 47-200 Kędzierzyn Koźle, Poland; 2 Department of Thoracic Surgery and Transplantation, Pomeranian Medical University in Szczecin, Alfreda Sokołowskiego 11, 70-891 Szczecin, Poland; 3 Department of Biochemistry and Medical Chemistry, Pomeranian Medical University, 70-111 Szczecin, Poland; 4 Clinical Department of Lung Diseases, Pomeranian Medical University in Szczecin, Alfreda Sokołowskiego 11, 70-891 Szczecin, Poland; 5 Clinic of General, Minimally Invasive and Gastroenterological Surgery, Pomeranian Medical University in Szczecin, Unii Lubelskiej 1, 71-252 Szczecin, Poland; 6 Clinic of Anesthesiology and Intensive Care, Pomeranian Medical University in Szczecin, Unii Lubelskiej 1, 71-252 Szczecin, Poland

**Keywords:** pneumonectomy, pulmonary hypertension, right atrium, right ventricle, pulmonary artery, pulmonary perfusion

## Abstract

**Background/Objectives**: After pneumonectomy, the right ventricular stroke volume is pumped into pulmonary vessels whose volume has been reduced by approximately 50%. To sustain conditions for pulmonary flow, the flow reserve is increased in the remaining lung, which is conducive to the development of pulmonary hypertension symptoms. This study sought to examine pulmonary flow in one lung and the size of the right atrium (RA), right ventricle (RV) and pulmonary artery (PA) in patients who had undergone pneumonectomy and to establish the influence of time since pneumonectomy on these parameters, as well as their potential mutual dependencies. **Methods**: The retrospective analysis included 34 patients who had undergone pneumonectomy. Pulmonary flow was measured by means of perfusion scintigraphy. The diameters of the RA, RV and PA were evaluated based on computed tomography with contrast. **Results**: We observed complete or near-complete utilization of flow reserve in 38.2% (13/34) of patients, enlarged transversal and longitudinal dimensions of the RA in 17.6% (6/34) and 32.3% (11/34) of patients, respectively, and enlarged transversal and longitudinal dimension of the RV in 67.6% (23/34) and 44.1% (15/34) of patients, respectively. Dilatation of the PA was discovered in 23.5% (8/34) to 26.5% (9/34) of patients, as well as the presence of an extensive complex of radiographic features of pulmonary hypertension (PH) syndrome in 23.5% (8/34) of cases. **Conclusions**: Radiological features of PH were present in a significant number of patients. These features developed at varying rates but were present in all patients followed >10 years after the procedure.

## 1. Introduction

Pneumonectomy is the most involved lung resection procedure, with anatomical and functional consequences. Removal of the lung activates the healing processes in the pleural cavity and chest wall on the thoracotomy side, frequently leading to the development of autothoracoplasty years later. The remaining lung is also subject to compensatory emphysema. In addition, the heart and the entire mediastinum shift to the operated side, with elevation and immobilization of the hemidiaphragm on the same side [1,2,3,4]. Another consequence of pneumonectomy is the loss of approximately 50% of the pulmonary circulation, which means that the entire stroke volume of the right ventricle of the heart (RV) flows into the reduced-capacity vascular system. Maintaining pulmonary flow conditions forces the use of flow reserves in the remaining lung with changes in the pulmonary artery (PA) system, favoring the development of symptoms of pulmonary hypertension (PH) secondary to the condition of the right heart chambers [1,5,6,7,8]. Therefore, changes in the flow system include broad functional aftereffects in the pulmonary circulation, including both the PA system and the right heart chambers. The present study examined selected parameters of pulmonary flow, PA status, and the right heart chambers in an available group of patients after pneumonectomy, with the aim of assessing the changes occurring and evaluating their severity.

## 2. Materials and Methods

### 2.1. Materials

The study group consisted of 34 patients who had undergone pneumonectomy and completed all research studies. One patient was operated on in 1972 and 33 patients in the period 1996–2019. The characteristics of the study group are presented in Table 1.

Ten patients were assessed within the first year after lung removal, 16 patients within 1–5 years after pneumonectomy, 4 patients within 5–10 years, and 4 patients > 10 years after surgery.

### 2.2. Methods

We analyzed the pulmonary flow and dimensions of the PA in the remaining lung and the dimensions of the RV and right atrium (RA) of the heart. The collection of the data had a retrospective character. The PA pressure measurements were not made using the Swan-Ganz catheter and transthoracic echocardiography due to their invasive nature or displacement of the mediastinum with a lack of effective and repeatable projection. Furthermore, there was no agreement on the use of transesophageal echocardiography. Therefore, the research plan was based on the availability of images obtained by computed tomography with contrast in the planar axial projections. The transverse and longitudinal, diameters of the RA, the transverse and longitudinal dimensions of the RV, the diameter of the PA after division into the right and left branch at the site of the first segmental branch, and the diameter of the main trunk of the PA (in its middle) were measured. All measurements were performed in the diastolic phase and at maximum luminal flow and reported in centimeters using the measurement tools of the e-Film and Exchibeon-3 systems. Pulmonary flow in the remaining lung was assessed by perfusion scintigraphy using human albumin macroaggregates labeled with the technetium (99mTc) radioisotope at a dose of 130–300 Bq administered intravenously 10 min before the examination according to the established protocol. The obtained results were processed using an MB 9200 Gamma Muvex Gamma camera with NMS software (*n* = 7 patients), AntScan Mediso Gamma camera with InterViewXP software (*n* = 11 patients), or Nucline AP Mediso Gamma camera with InterViewXP software (*n* = 16 patients). The results were standardized for further evaluation and analysis. We quantified perfusion (% perfusion) in each of three (upper, middle, and lower) equal fields of the single lung. We also performed an analysis of the upper field perfusion as an indicator of the utilization of flow reserves in the lung. Perfusion was assessed at different times after pneumonectomy in the stabilized flow phase. Patients with lesions in a single lung changing pulmonary blood flow and patients with additional cardiac failure were excluded from the study. The time interval from pneumonectomy to scintigraphic examination ranged from 9 days to 16,469 days (average 1785.4 days).

### 2.3. Cases

The study group consisted of both uncomplicated and postpneumonectomy empyema (PPE) cases. All of the PPE cases were in stable functional status and treatment did not affect pulmonary circulation or the anatomy of the chest on the opposite side to the pneumonectomy. There were no cases with active bronchopleural fistula, but 4 patients were receiving permanent treatment with drainage or thoracostomy. The measurements in the entire study group were analyzed in terms of the side of the operation, the time interval from pneumonectomy, and the size of the PA and right heart chambers. The variables were presented as means and standard deviations. The level of significance was assumed to be *p* < 0.05.

## 3. Results

Lung field flow values are given in Table 2 and Table 3. In 50% (17/34) of cases, the flow in the upper field was greater than the average upper field perfusion of 21.9%. In 38.2% (13/34) of patients, the flow ranged from 23.3% to 28.5%, meeting the criteria for high (23.3–24.8%) and almost full or full use (25–28.5%) of flow reserves [9,10,11,12,13,14,15].

In 53% (18/34) of patients, the transverse dimension of the RA was equal to or greater than the average value of 4 cm. In 17.6% (6/34) of patients, the transverse dimension of the RA was in the range of 4.3–6 cm, indicating its widening (>4.3 cm) [16]. In 47% (16/34) of patients, the longitudinal dimension of the RA was greater than the mean value of 5.2 cm. In 32.3% (11/34) of patients, the longitudinal dimension of the RA was within the range indicating its widening (5.7–6.4 cm; reference value < 5.4 cm) [16]. In 50% (17/34) of patients, the transverse dimension of the RV was greater than the mean value of 4.3 cm. However, in 64.7% (22/34) of patients, the transverse dimension of the RV was within the range indicating its widening (4.2–5.2 cm; reference value < 4.1 cm) [17]. The longitudinal dimension of the RV was greater than the average value of 8.5 cm in 44.1% (15/34) of patients [17]. In this subgroup, the longitudinal dimension of the RV was in the range of 8.6–11 cm, indicative of its widening (reference value < 8.3 cm) [17].

Furthermore, in the analysis of the surgical side, we found significant differences in the longitudinal dimensions of the RA and RV depending on side of operation. The patients with the remaining left lung had a significantly larger longitudinal dimension of the RA compared to the patients with the remaining right lung (5.7 cm vs. 4.7 cm; *p* = 0.0001). There was also a significantly larger longitudinal dimension of the RV in patients with the remaining right lung compared to patients with the remaining left lung (9 cm vs. 8 cm; *p* = 0.025). In 32.35% (11/34) of patients, the diameter of the PA behind its main division (PA I) was greater than the mean value of 2.4 cm. In 26.5% (9/34) of patients, PA I met the criteria for extension (>2.51 cm) [18]. In the other two patients with PA I > 2.4 cm, the diameter almost reached 2.5 cm. In 35.3% (12/34) of patients, the diameter at the site of the first segmental branch (PA II) was greater than the mean value of 2.2 cm. In 23.5% (8/34) of patients PA II was within the range indicating its widening (2.4–2.8 cm; reference value > 2.3 cm). In 44.1% (15/34) of patients, the diameter of the PA at the level of the main trunk (PA III) was greater than the mean value of 2.8 cm; 26.5% (9/34) of patients met the criteria for dilatation (3–3.5 cm; reference value > 2.95 cm). Furthermore, in 17.6% (6/34) of patients, the diameter of the PA had already reached the limiting dimension of 2.9 cm [19]. The individual dimensions are given in Table 4 and Table 5.

**Figure 1:**
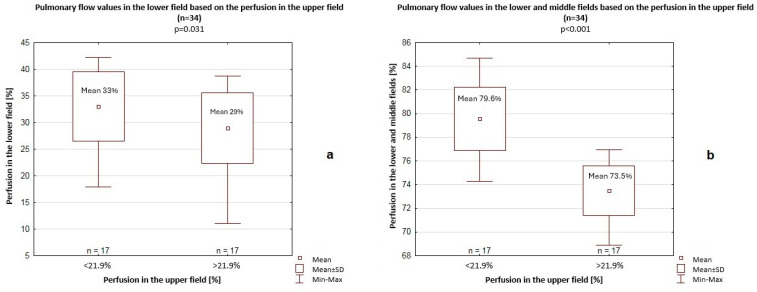
Pulmonary flow values in the lower field (**a**) or in the lower and middle fields (**b**) based on the perfusion in the upper field. SD—standard deviation.

The mean pulmonary perfusion in the upper field was 21.9%. Among patients with >21.9% perfusion in the upper field, significantly lower pulmonary flow values were recorded in the lower field (29% vs. 33%, *p* = 0.031) and in the lower and middle fields combined (73.5% vs. 79.6%, *p* < 0.001) compared to patients with <21.9% perfusion (Figure 1).

**Figure 2:**
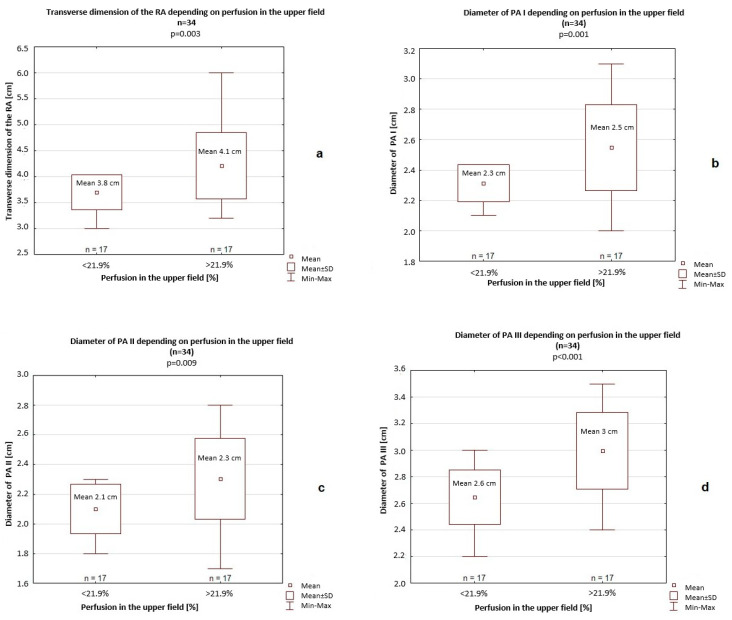
The measured transverse dimension of the right atrium (**a**) and diameters of pulmonary artery (PA) I (**b**), PA II (**c**), and PA III (**d**) depending on perfusion in the upper field. SD—standard deviation.

In the group with pulmonary flow in the upper field > 21.9%, the transverse dimension of the RA (mean 4.1 cm vs. 3.8 cm, *p* = 0.003) and all dimensions of the PA were significantly greater (PA I: 2.5 cm vs. 2.3 cm, *p* = 0.001; PA II: 2.3 cm vs. 2.1 cm, *p* = 0.008; PA III: 3 cm vs. 2.6 cm, *p* < 0.001; Figure 2).

**Figure 3:**
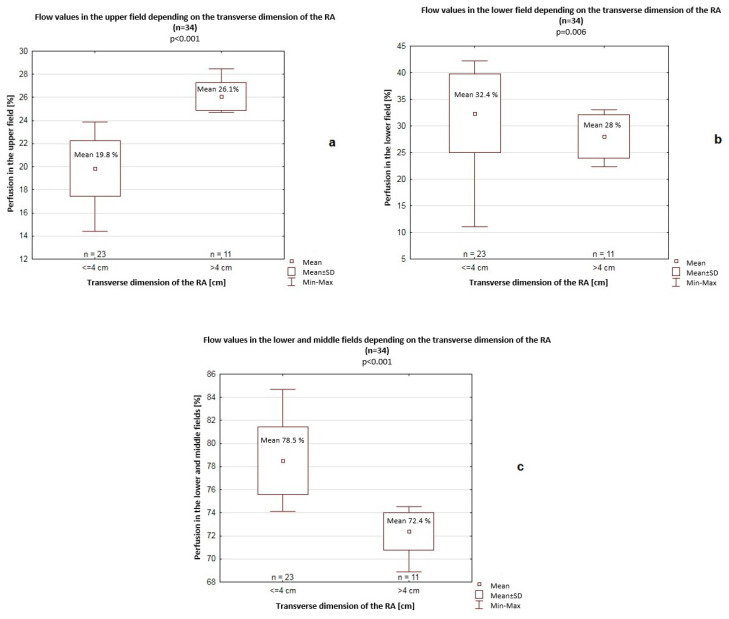
Flow values in the upper field (**a**), lower field (**b**), and lower and middle fields (**c**) depending on the transverse dimension of the right atrium (RA). SD—standard deviation.

The transverse dimension of the RA was 4 cm on average. Among patients with a >4 cm transverse dimension of the RA, significantly higher perfusion values were recorded in the upper field (26.1% vs. 19.8%, *p* < 0.001) but significantly lower perfusion values in the lower field (28% vs. 32.4%, *p* = 0.006) and in the lower and middle fields (72.4% vs. 78.5%, *p* < 0.001) compared to patients with a <4 cm transverse dimension of the RA (Figure 3).

**Figure 4:**
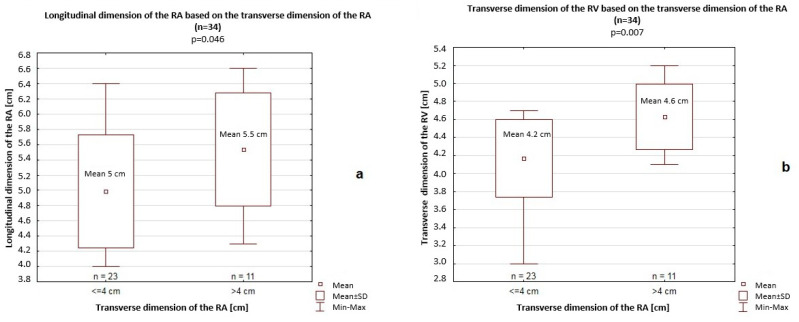
Longitudinal dimension of the right atrium (RA) (**a**) and transverse dimension of the right ventricle (RV) (**b**) based on the transverse dimension of the RA. SD—standard deviation.

**Figure 5:**
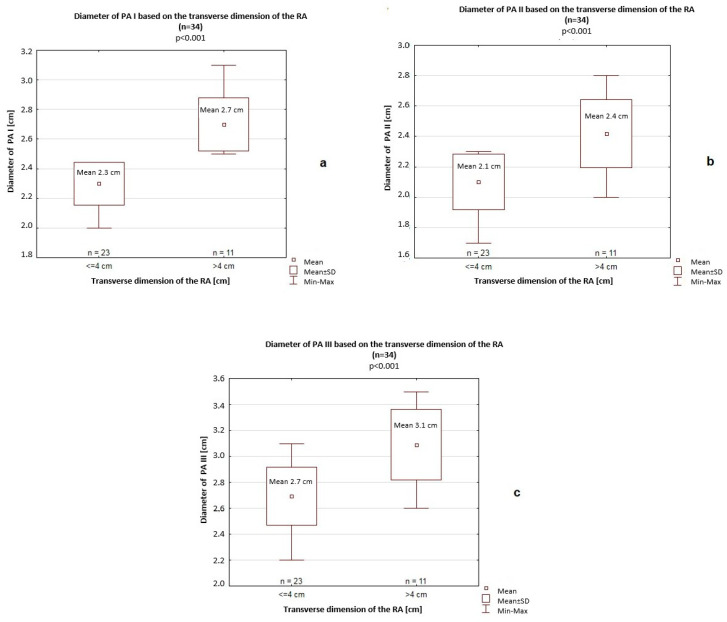
Diameters of the pulmonary artery (PA) based on the transverse dimension of the right atrium (RA). (**a**) PA I, (**b**) PA II, and (**c**) PA III. SD, standard deviation.

In this comparison, a significantly greater longitudinal dimension of the RA (5.5 cm vs. 5 cm, *p* = 0.046) and transverse dimension of the RV (4.6 cm vs. 4.2 cm, *p* = 0.007) were also noted (Figure 4). Furthermore, all PA diameters (PA I–III) were significantly greater among patients with a >4 cm transverse dimension of the RA (PA I: 2.7 cm vs. 2.3 cm, *p* < 0.001; PA II: 2.4 cm vs. 2.1 cm, *p* < 0.001; PA III: 3.1 cm vs. 2.7 cm, *p* < 0.001) compared to patients with a <4 cm transverse dimension of the RA (Figure 5). Differences in the longitudinal dimension of the RV based on the transverse dimension of the RA (>4 cm vs. <4 cm) were not significant (8.4 cm vs. 8.8 cm; *p* = 0.46).

**Figure 6:**
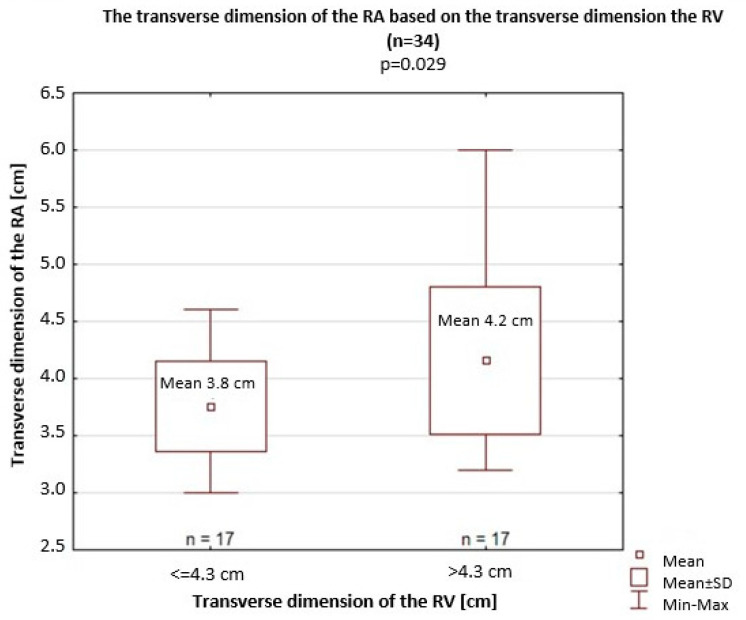
The transverse dimension of the right atrium (RA) based on the transverse dimension of the right ventricle (RV). SD, standard deviation.

The transverse dimension of the RV was 4.3 cm on average. Among patients with a >4.3 cm transverse dimension of the RV, a significantly greater transverse dimension of the RA was recorded (4.2 cm vs. 3.8 cm; *p* = 0.029) than among patients with a <4.3 cm transverse dimension of the RV (Figure 6). In this subgroup, the differences in terms of upper field perfusion (22.07% vs. 21.7%; *p* = 0.70), PA I (2.5 cm vs. 2.4 cm; *p* = 0.099), PA II (2.3 cm vs. 2.1 cm; *p* = 0.12), and PA III (2.9 cm vs. 2.7 cm; *p* = 0.09), and the longitudinal dimension of the RV (8.1 cm vs. 8.9 cm; *p* = 0.09) did not reach significance.

The longitudinal dimension of the RV was 8.5 cm on average. There were no significant differences in the transverse dimension of the RV (4.2 cm vs. 4.5 cm, *p* = 0.12) or any of the dimensions of the RA (transverse: 3.9 cm vs. 4 cm, *p* = 0.31; longitudinal: 5.2 cm vs. 5.1 cm, *p* = 0.6) or PA (PA I: 2.4 cm vs. 2.5 cm, *p* = 0.11; PA II: 2.1 cm vs. 2.3 cm, *p* = 0.19; PA III: 2.7 cm vs. 2.9 cm, *p* = 0.08) based on the longitudinal dimension of the RV being < 8.5 cm or >8.5 cm. However, we noted a significant difference related to weight (67.5 kg vs. 79.5 kg, *p* = 0.03) and BMI (23.7 vs. 27.6, *p* = 0.03).

**Figure 7:**
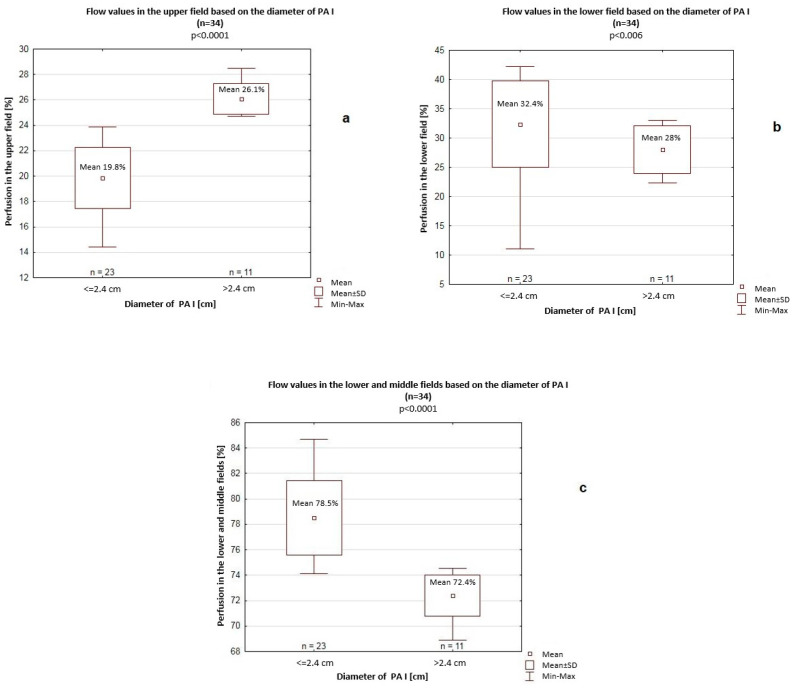
Flow values in the upper field (**a**), lower field (**b**), and lower and middle fields (**c**) based on PA I. PA I, the diameter of the pulmonary artery at the division of the right and left branches. SD—standard deviation.

The comparison of the diameters of the PA was performed based on the diameter of PA I, which was 2.4 cm on average. Among patients with a PA I > 2.4 cm, significantly greater pulmonary flow was observed in the upper field (26.1% vs. 19.8%, *p* < 0.001) and significantly lower pulmonary perfusion in the lower field (28% vs. 32.4%, *p* = 0.006) and in the lower and middle fields (72.4% vs. 78.5%, *p* < 0.001; Figure 7).

**Figure 8:**
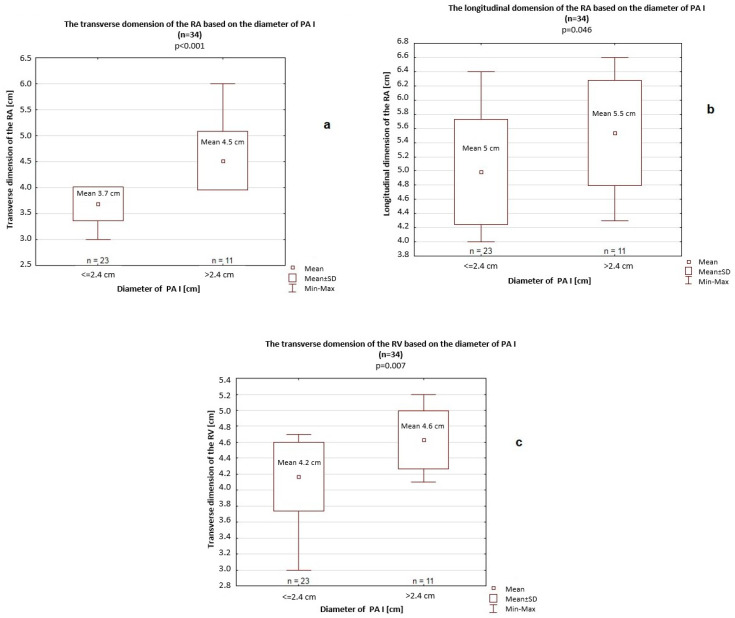
Dimensions of the right atrium (RA) and right ventricle (RV) based on PA I. (**a**) Transverse dimension of the RA. (**b**) Longitudinal dimension of the RA. (**c**) Transverse dimension of the RV. PA I, the diameter of the pulmonary artery at the division of the right and left branches. SD—standard deviation.

**Figure 9:**
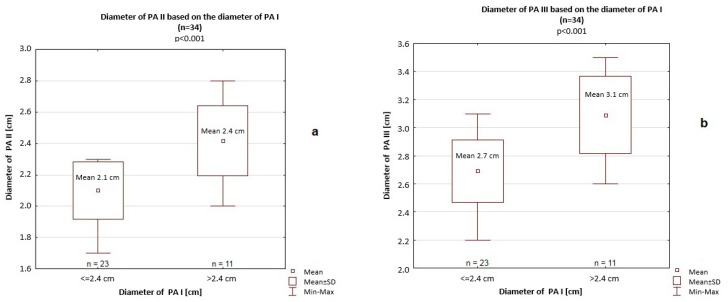
PA II (**a**) and PA III (**b**) based on PA I. PA I, diameter of the pulmonary artery at the division of the right and left branches; PA II, diameter of the pulmonary artery at the site of the first segmental branch; PA III, diameter of the pulmonary artery at the level of the main trunk. SD—standard deviation.

Among patients with a PA I > 2.4 cm, the transverse and longitudinal dimensions of the RA were significantly greater (4.5 cm vs. 3.7 cm, *p* < 0.001 and 5.5 cm vs. 5 cm, *p* = 0.046, respectively), as was the transverse dimension of the RV (4.6 cm vs. 4.2 cm, *p* = 0.007) compared to patients with a PA I < 2.4 cm (Figure 8). In this subgroup (PA I > 2.4 cm), significantly greater PA II (2.4 cm vs. 2.1 cm, *p* < 0.001) and PA III (3.1 cm vs. 2.7 cm, *p* < 0.001) measurements were also observed (Figure 9), but we did not find significant differences in the longitudinal dimension of the RV (8.4 cm vs. 8.8 cm, *p* = 0.46).

We also found numerous positive and negative correlations between the above-mentioned features, which are outlined in Table 6 and Table 7.

We found positive correlations between pulmonary perfusion in the upper field, PA diameter, and transverse dimensions of the RA and RV consistent with the state of and changes in the pulmonary circulation. The negative correlations we identified concerned opposing perfusion features in individual lung fields and the related dimensions of the PA. The above relationships excluded the longitudinal dimension of the RV, which correlated with BMI and weight.

The absence of features of PH or the presence of a single feature of PH was observed in 8 patients. A set of at least five coexisting features of PH was found in another 8 patients. However, the majority of patients (*n* = 18) had two to four features of PH. A summary of the analyzed features in relation to literature standards depending on the time from pneumonectomy is presented in Table 8, Table 9, Table 10 and Table 11.

An analysis of the relationship between the results and published norms among observations made up to 1 year after pneumonectomy revealed that 60% of cases were within the accepted limits or exceeded the norms for only a single feature (0 to 1), whereas 40% of cases had three to eight coexisting features of PH.

The observations made 1–5 years after pneumonectomy revealed that 37.5% of cases exceeded the norms for one to two features of PH, and 62.5% exceeded the norms for three to eight coexisting features of PH (Table 9).

In the period 5–10 years from pneumonectomy, 75% of cases either fell within the accepted limits or exceeded the norms for zero to two features, and in 25% of cases a syndrome of three coexisting features of PH was observed (Table 10).

The observations made >10 years after pneumonectomy revealed four to seven coexisting features of PH in all patients. However, there were no physiological relationships between the analyzed features of the pulmonary circulatory system or with individual PH features. The above trend does not include the longitudinal dimension of the RV, as widening occurred in only one case (Table 11).

## 4. Discussion

Stabilization of respiratory function after pneumonectomy is accompanied by an increase in the flow in the remaining lung, remodeling of the circulatory system, including both the PA and right heart cavities, and subsequent development of features of PH syndrome [1,5,6,7,20,21,22,23,24]. This is attributed to the use of flow reserves in the remaining lung to stabilize the PA pressure, but elevated systolic pressure in the RV with features of mild PH have been reported on the second day after removal of an entire lung [25]. Increased flow in the one-lung system is accompanied by an increase in pressure in the PA and leads to dilatation of the right heart cavities and the remaining branch of the PA as a cascade of postoperative events [1,5,6,7,8,20,26,27]. Lung circulation changes involve the use of previously inactive areas within the pulmonary capillaries, which is particularly evident in the upper fields of the remaining lung [9,10,11,23]. Perfusion of the single upper field should not exceed 10% of the total lung perfusion in a two-lung system [9,10,11,12]. Previous studies noted 21.35% perfusion after pneumonectomy [11]. In the current study, the average perfusion level of the upper field was 21.9%, which is consistent with previous observations. The importance of the level of upper field perfusion is that increasing the flow from <10% in the two-lung system to ≥21.9% in the one-lung system intensively uses the lung flow reserves; after exceeding 25%, it practically exhausts them as a mechanism for stabilizing the pulmonary flow and counteracting PH [28]. This situation concerned 13 patients in the study group with submaximal or maximal flow in the range of 23.3–28.5%, particularly 9 patients with flow in the range of 25–28.5%. According to Chandra, increased perfusion in the upper field is associated with an unfavorable prognosis, and the limiting value is 20% of flow [13]. Another phenomenon that was observed when the perfusion of the upper field increased was a proportional decrease in perfusion in the other fields of the remaining lung as one common flow area. This observation is important in regard to changes in the ventilation–perfusion (V/Q) ratio and the effectiveness of the blood oxygenation process [29].

The mean diameter of the PA at the point of division into the right and left branches (i.e., PA I) was 2.4 cm. One-third of the patients in the study group had a PA I > 2.4 cm, and more than one-fourth had a PA I greater than the reference value of 2.51 cm [18]. The mean PA diameter at the level of the main trunk (i.e., PA III) was 2.8 cm. Nearly half of the patients in the study group had a PA III > 2.8 cm, and more than one-fourth had a PA III greater than the reference value of 29.5 cm [19,30]. This observation has an additional, and at the same time unfavorable, importance; according to Żyłkowska, an enlarged PA diameter is recognized as an independent risk factor for death in patients with PH [31]. No data were found in the literature regarding the PA diameter at the site of the first segmental branch (i.e., PA II). The obtained average of 2.2 cm may be the basis for future comparisons and analyses.

The mean transverse and longitudinal dimensions of the RA in the study group were 4 cm and 5.2 cm, respectively. According to Maceira, the reference values for mean RA dimensions for the European population are 4.3 cm and 5.4 cm, respectively [16]. More than half (53%) of the patients in the study group had a transverse dimension of the RA ≥ 4 cm, and nearly half (47%) of the patients had a longitudinal dimension of the RA > 5.2 cm. In Maceira’s data, nearly one-sixth (17.6%) of the RA transverse dimensions and nearly one-third (32.3%) of the RA longitudinal dimensions were greater than the cited reference dimensions. A relationship between RA changes and PH was described by Alenezi [32]. Moreover, enlargement, and even dysfunction, of the RA was reported in a group of patients with PH [33,34], and a positive correlation between increased systolic pressure in the PA and RA dilatation in patients with PH was observed by Cioffi [35]. According to the literature, enlargement of the dimensions of the RA and PA is associated with the development of PH and with potential worsening of the prognosis [31,32,33,35,36,37,38,39,40,41]. Other negative prognostic factors in pulmonary hypertension include signs of right heart failure, frequent syncope, elevated pulmonary vascular resistance, elevated right atrial pressure, reduced tricuspid annular plane systolic excursion (TAPSE), tricuspid regurgitation, pericardial effusion, reduced right ventricular ejection fraction, and decreased right ventricular septal and global longitudinal strain [42,43,44,45].

Furthermore, RV dilatation after pneumonectomy has been reported in the literature [6,7,20,26,46,47]. Kowalewski demonstrated an increase in the dimensions and deterioration of the systolic function of the RV 2 days after pneumonectomy, which was confirmed in subsequent studies [5,7,25,48]. The particular value of analyzing the dimensions of the RV lies in the combination of its dilated morphology and impairment of its systolic function, though the efficiency of the RV was not examined in the current study [7,8,25,38,47,48,49,50]. The relationship between increased flow in the remaining lung, increased pressure and size in the PA, and dilatation of the right chambers of the heart was also described by Foroulis and Venuta [1,5,6,7,8,20,26]. Interestingly, the ratio of TAPSE and systolic pulmonary arterial pressure (sPAP) was introduced as a marker of the coupling of pulmonary circulation and right ventricle [51]. Moreover Tello et al. demonstrated the prognostic value of this parameter in PH patients. Lower TAPSE/sPAP ratio was associated with worse overall survival [52]. Sonaglioni et al. also demonstrated the predictive value of TAPSE/sPAP ratio in patients with idiopathic pulmonary fibrosis [53]. Unfortunately, we did not measure this parameter in our study. The mean transverse and longitudinal dimensions of the RV in the current study were 4.3 cm and 8.5 cm, respectively. In the NORRE study, the reference dimensions of the RV were 3.4 cm and 6.8 cm, respectively, but the physiological transverse dimension could reach 4.1 cm and the longitudinal dimension up to 8.3 cm [54]. Błażejewski in his analysis reported maximum normal RV basal and longitudinal diameters in accordance with American and European Echocardiographic Guidelines (4.1 cm and 8.3 cm, respectively) [17]. Almost all patients were characterized by a larger transverse and longitudinal dimension of the RV than the cited standards. However, differences in imaging techniques should be taken into account when interpreting these results. This selective interpretation also applies to the current study and constitutes its limitation.

The relationships observed in the pulmonary circulation are closely related, with a statistical correlation between the level of pulmonary flow, the dimensions of the PA, and most of the dimensions of the right heart cavities (see Table 6 and Table 7). Perfusion in the upper field significantly positively correlated with all sizes of PA I, PA II, and PA III and significantly negatively correlated with the perfusion in the lower field and perfusion in the combined lower and middle fields. In the group of patients with PA I > 2.4 cm, significantly greater transverse and longitudinal dimensions of the RA, transverse dimension of the RV, and other dimensions of the PA (i.e., PA II and PA III) were demonstrated (Figure 8 and Figure 9). The reported relationships are based on earlier publications by Żyłkowska, Ratanawatkul, and Oganesian [15,30,31]. Among patients with a transverse dimension of the RA > 4 cm, significantly higher perfusion was observed in the upper field of the remaining lung with significantly lower flow in the lower field and in the combined lower and middle fields. In this group of patients, significant enlargement of the longitudinal dimension of the RA, transverse dimension of the RV, and all three diameters of the PA were also noted. The association of PH with RA dilatation and increased systolic pressure in the PA in patients with PH was reported by Cioffi, and the association of an increase in PA diameter, systolic pressure, and RV systolic functional status in patients with PH was reported by Tonelli [35,36]. The transverse dimension of the RV is part of these relationships. For patients with a transverse dimension of the RV > 4.3 cm, significantly greater transverse dimensions of the RA were found. In addition, the value of the transverse dimension of the RV significantly positively correlated with all diameters of the PA. There are many studies in the literature on PH regarding RV augmentation [6,7,20,26,45,55]. Fisher reported a positive correlation of systolic pressure in the PA and vascular resistance with RA and RV enlargement in patients with PH [38]. Enlargement of the RV diameter, muscle wall area, and outflow tract in a group of patients with PH was also described by Swift et al. [56]. However, we did not identify a statistical correlation between the longitudinal dimension of the RA and pulmonary perfusion in the upper field or PA I. Another type of relationship was observed for the longitudinal dimension of the RV, significant positive correlations with weight and BMI. The lack of other relationships for the longitudinal dimension of the RV may indicate an asymmetric influence of the two dimensions of the RV and, in some cases, the predominant influence of the transverse dimension in the process of RV dilatation.

The majority of studies have described changes in the immediate period after lung removal and in the first 5 years after pneumonectomy [7,20,25,26,48]. The condition of patients > 5 years after pneumonectomy was documented by Deslauries [5], but longer term observations have been rare. The literature indicates that the process of PH development does not proceed via the same dynamics in all patients. It seems that the characteristics of this process were most accurately presented by Potaris, who demonstrated an increase in systolic pressure in the PA and the development of PH in nearly 40% of patients within the first year after pneumonectomy [57]. The results obtained in the present study were similar to previous observations. The flow reserves in the lung were used or exceeded in almost 40% of patients. Dilatation of the right heart chambers occurred in 17.6–64.7% of patients depending on the size examined, as well as in 23.5–26.5% of patients when analyzing PA diameter. PH symptoms were present in 40% of patients already in the first year of follow-up, 62.5% of patients 1–5 years after pneumonectomy, 25% of patients 5–10 years after pneumonectomy, and all patients who were followed >10 years after the procedure. Moreover, a severe set of PH features was detected in nearly a quarter of cases.

The results of the present study indicate that, in the adopted model of increased perfusion in the upper field of a single lung, dilatation of the RA, RV, and PA develops at a different rate during the first 10 years after the procedure. However, the small group size limits our conclusions. A similar situation and limitations will apply to the global dimension for cases requiring removal of the entire lung for oncological reasons [4].

## 5. Conclusions

After pneumonectomy, radiological features of PH were observed in a significant percentage of cases. Features of PH developed at a different pace in subsequent years of observation but were present in all patients followed >10 years after the procedure. The pulmonary circulatory system limited to the remaining lung after pneumonectomy results in significant relationships between the degree of pulmonary perfusion, the size of the RA and RV, and the diameter of the PA. However, correlations with respect to the longitudinal dimension of the RV were not demonstrated.

## Figures and Tables

**Table 1:** Characteristics of the study group.

Number of Patients in the Study Group	34
Male, *n*	27
Female, *n*	7
Age range, years	47–78
Average age, years ± SD	62.6 ± 7.9
Operated side—right, *n*	16
Operated side—left, *n*	18
Left ventricular ejection fraction range, %	41–68
Average left ventricular ejection fraction, % ± SD	54.8 ± 6.7
Operated for oncological reasons, *n*	32
Operated for non-oncological reasons, *n*	2
Uncomplicated course after pneumonectomy, *n*	14
Pleural empyema after pneumonectomy, *n*	20
Cured of pleural empyema, *n*/*n*_c_ (%) ^a^	16/20 (80%)
Drain carriers/permanent thoracostomy, [*n*/*n*_c_] (%)	4/20 (20%)
Time range from pneumonectomy to scintigraphy, days	9–16,469
Average time from pneumonectomy to scintigraphy, days ± SD	1785.4 ± 3099.1
Height range, cm	158–196
Average height, cm± SD	169.4 ± 8.7
Weight range, kg	42.6–108
Average weight, kg ± SD	72.8 ± 15.3
BMI range, kg/m^2^	15.4–35.9
Average BMI, kg/m^2^ ± SD	25.4 ± 5.2

Abbreviations: SD, standard deviation; BMI, body mass index. ^a^ The ratio of the number of cases to the whole group.

**Table 2:** Flow values in individual fields in the right lung (*n* = 18).

Patient	Flow in the Upper Field, %	Flow in the Middle Field, %	Flow in the Bottom Field, %	Flow in the Middle and Bottom Fields, %
1	19.9	47.8	32.3	80.1
2	16.6	49.0	34.2	83.2
3	17.8	49.0	33.0	82.0
4	26.1	40.5	31.3	71.8
5	18.4	62.3	17.9	80.3
6	19.6	40	38.3	78.3
7	24.8	44.5	28.2	72.7
8	17.9	43.5	36.3	79.8
9	25.0	43.0	29.3	72.3
10	18.6	41.9	36.2	78.1
11	22.3	40.7	35.7	76.4
12	22.4	36.1	38.7	74.8
13	22.8	40.3	34.8	75.1
14	21.3	42.7	35.4	78.1
15	20.9	45.5	29.0	74.5
16	24.7	41.1	33.0	74.1
17	27.0	38.3	32.9	71.2
18	25.3	43.1	30.8	73.9

**Table 3:** Flow values in individual fields in the left lung (*n* = 16).

Patient	Flow in the Upper Field, %	Flow in the Middle Field, %	Flow in the Bottom Field, %	Flow in the Middle and Bottom Fields, %
1	21.2	49.7	23.1	72.8
2	20.7	60.1	18.8	78.8
3	22.4	65.9	11.0	77.0
4	25.4	48.9	25.7	74.6
5	23.9	40.6	33.5	74.1
6	19.2	50.8	30.0	80.7
7	23.3	45.7	30.1	75.8
8	21.1	44.1	30.2	74.3
9	28.5	46.6	22.3	68.9
10	27.1	48.7	22.3	71.0
11	25.8	43.4	29.5	72.9
12	14.4	42.5	42.2	84.7
13	17.8	44.4	36.9	81.3
14	17.9	41.0	39.2	80.2
15	17.3	44.2	36.0	80.2
16	20.1	42.9	35.3	78.2

**Table 4:** Dimensions of the right ventricle, right atrium, and pulmonary artery in patients in whom the right lung was assessed (*n* = 18).

Patient	Diameter RA, cm	Length RA, cm	Diameter RV, cm	Length RV, cm	DiameterPA I, cm	DiameterPA II, cm	DiameterPA III, cm
1	4	5.5	4.3	7.6	2.4	2.2	2.6
2	3.9	4.2	4.7	8.7	2.4	2.2	2.6
3	4	4	4.1	7.6	2.4	2.3	2.5
4	4.3	4.3	4.6	7.5	2.6	2.2	3
5	3.9	4.8	4.6	10	2.4	2.2	2.9
6	4	4.4	4.6	7.5	2.4	2.2	2.5
7	4.1	5.1	5.2	11	2.7	2.4	2.9
8	3.9	4.5	3.8	9	2.3	1.9	3
9	4.6	4.9	4.9	10.6	3.1	2.8	3.3
10	4	4.7	4.7	10.4	2.4	2.3	2.7
11	3.9	4.3	4	10.4	2.4	2.2	2.9
12	3.8	4.3	4	10.5	2.4	2.2	2.9
13	3.2	4.3	4.7	8.2	2	1.7	2.7
14	3.2	5	4.5	7.3	2.1	1.8	2.8
15	3.4	4.3	3.8	8.2	2.2	2	2.5
16	4.4	6	4.4	8.6	2.6	2.4	3.3
17	4.2	4.8	4.1	9.8	2.6	2.2	2.9
18	4.2	5.1	4.7	8.6	2.7	2.6	2.8

Abbreviations: Diameter RA, transverse dimension of the right atrium of the heart; Length RA, longitudinal dimension of the right atrium of the heart; Diameter RV, transverse dimension of the right ventricle of the heart; Length RV, longitudinal dimension of the right ventricle of the heart; Diameter PA I, diameter of the pulmonary artery behind the division into the right and left branches; Diameter PA II, diameter of the pulmonary artery at the site of donation I segmental branch; Diameter PA III, the diameter of the pulmonary artery in the middle part of the main trunk.

**Table 5:** Dimensions of the right ventricle, right atrium, and pulmonary artery in patients in whom the left lung was assessed (*n* = 16).

Patient	Diameter RA, cm	Length RA, cm	Diameter RV, cm	Length RV, cm	Diameter PA I, cm	Diameter PA II, cm	Diameter PA III, cm
1	4.1	5.6	4.3	8	2.7	2.5	3.1
2	3.5	4	3	8	2.1	1.9	2.2
3	3.4	5.7	4	7	2	1.9	2.4
4	4.2	6.6	5	10.5	2.8	2.5	3.5
5	3.6	4.4	3.6	6.5	2.4	2.3	3.1
6	3.3	5.5	4	10.3	2.4	2.2	2.7
7	4	5.5	4.3	6.1	2.4	2.3	2.9
8	3	6	4.2	8.2	2.4	2.2	2.7
9	5	6	4.5	7.3	2.5	2.4	3.3
10	4.6	6.1	4.2	7	2.5	2	2.6
11	6	6.4	5	7.6	2.9	2.6	3.3
12	3.7	5.5	4.5	10	2.1	1.8	2.6
13	4	6	4.4	10.1	2.4	2.2	2.8
14	3.4	5.7	3.6	7	2.2	2	2.3
15	3.7	5.7	4	6.2	2.3	2.1	.8
16	4	6.4	.5	7.8	2.4	2.2	2.8

Abbreviations: Diameter RA, transverse dimension of the right atrium of the heart; Length RA, longitudinal dimension of the right atrium of the heart; Diameter RV, transverse dimension of the right ventricle of the heart; Length RV, longitudinal dimension of the right ventricle of the heart; Diameter PA I, diameter of the pulmonary artery behind the division into the right and left branches; Diameter PA II, diameter of the pulmonary artery at the site of donation I segmental branch; Diameter PA III, the diameter of the pulmonary artery in the middle part of the main trunk.

**Table 6:** Positive correlations identified in individual fields in the study group.

Trait I	Trait II	Spearman’s Rank Correlation Coefficient	*p*
Transverse dimension RA	Pulmonary perfusion in the upper field	0.57	0.0003
Transverse dimension RA	PA I	0.85	<0.0001
PA I	Pulmonary perfusion in the upper field	0.65	<0.0001
PA II	Pulmonary perfusion in the upper field	0.47	0.004
PA III	Pulmonary perfusion in the upper field	0.57	0.0003
Transverse dimension RV	PA I	0.52	0.001
Transverse dimension RV	PA II	0.47	0.004
Transverse dimension RV	PA III	0.34	0.043
PA I	PA II	0.86	<0.0001
PA I	PA III	0.67	<0.0001
Transverse dimension RA	Transverse dimension RV	0.51	0.001
Weight	Longitudinal dimension RV	0.45	0.006
BMI	Longitudinal dimension RV	0.41	0.013
Longitudinal dimension RV	Pulmonary perfusion in the upper field	−0.073	0.67
Longitudinal dimension RV	PA I	0.28	0.1
Longitudinal dimension RV	Transverse dimension RV	0.33	0.055

Abbreviations: RA, right atrium; RV, right ventricle; PA I–III, pulmonary artery diameters.

**Table 7:** Negative correlations identified in individual fields in the study group.

Trait I	Trait II	Spearman’s Rank Correlation Coefficient	*p*
Pulmonary perfusion in the upper field	Pulmonary perfusion in the lower field	−0.55	0.0006
Pulmonary perfusion in the upper field	Pulmonary perfusion in the lower and muddle field	−0.94	<0.0001
Pulmonary perfusion in the lower field	Pulmonary perfusion in the middle field	−0.67	<0.0001
PA I	Pulmonary perfusion in the lower and muddle fields	−0.62	<0.0001
PA II	Pulmonary perfusion in the lower and muddle fields	−0.44	0.007
PA III	Pulmonary perfusion in the lower and muddle fields	−0.54	0.0007

Abbreviations: RA, right atrium; RV, right ventricle; PA I–III, pulmonary artery diameters.

**Table 8:** Comparison of pulmonary perfusion in the upper field, transverse and longitudinal dimensions of the right atrium and right ventricle, and the diameter of the pulmonary artery at individual measurement points relative to the literature standards up to 1 year after pneumonectomy.

Patient	Perfusion, ^a^ %	Diameter RA, cm	Length RA, cm	Diameter RV, cm	Length RV, cm	Diameter PA I, cm	Diameter PA II, cm	Diameter PA III, cm
1	>23.3	>4.3	<5.4	>4	<8.3	>2.51	<2.2	>2.95
2	<23.3	<4.3	<5.4	>4	<8.3	<2.51	<2.2	<2.95
3	>23.3	<4.3	<5.4	>4	>8.3	>2.51	>2.2	<2.95
4	<23.3	<4.3	<5.4	<4	>8.3	<2.51	<2.2	<2.95
5	<23.3	<4.3	<5.4	<4	>8.3	<2.51	<2.2	<2.95
6	<23.3	<4.3	<5.4	>4	<8.3	<2.51	<2.2	<2.95
7	<23.3	<4.3	<5.4	<4	<8.3	<2.51	<2.2	<2.95
8	>23.3	>4.3	>5.4	>4	>8.3	>2.51	>2.2	>2.95
9	<23.3	<4.3	>5.4	<4	<8.3	<2.51	<2.2	<2.95
10	<23.3	<4.3	>5.4	>4	>8.3	<2.51	<2.2	<2.95

Abbreviations: Diameter RA, transverse dimension of the right atrium of the heart; Length RA, longitudinal dimension of the right atrium of the heart; Diameter RV, transverse dimension of the right ventricle of the heart; Length RV, longitudinal dimension of the right ventricle of the heart; Diameter PA I, diameter of the pulmonary artery behind the division into the right and left branches; Diameter PA II, diameter of the pulmonary artery at the site of donation I segmental branch; Diameter PA III, the diameter of the pulmonary artery in the middle part of the main trunk. ^a^ Pulmonary perfusion in the upper field.

**Table 9:** Comparison of pulmonary perfusion in the upper field, transverse and longitudinal dimensions of the right atrium and right ventricle, and the diameter of the pulmonary artery at individual measurement points relative to the literature standards 1–5 years after pneumonectomy.

Patient	Perfusion, ^a^ %	Diameter RA, cm	Length RA, cm	Diameter RV, cm	Length RV, cm	Diameter PA I, cm	Diameter PA II, cm	Diameter PA III, cm
1	<23.3	<4.3	<5.4	>4	<8.3	<2.51	>2.2	<2.95
2	<23.3	<4.3	<5.4	>4	>8.3	<2.51	<2.2	<2.95
3	<23.3	<4.3	<5.4	<4	>8.3	<2.51	<2.2	>2.95
4	>23.3	<4.3	<5.4	>4	>8.3	>2.51	>2.2	>2.95
5	<23.3	<4.3	<5.4	>4	>8.3	<2.51	>2.2	<2.95
6	<23.3	<4.3	<5.4	>4	<8.3	<2.51	<2.2	<2.95
7	>23.3	<4.3	<5.4	>4	>8.3	>2.51	<2.2	<2.95
8	>23.3	>4.3	>5.4	>4	<8.3	>2.51	>2.2	>2.95
9	>23.3	>4.3	>5.4	>4	>8.3	>2.51	>2.2	>2.95
10	>23.3	<4.3	<5.4	<4	<8.3	<2.51	>2.2	>2.95
11	<23.3	>4.3	>5.4	<4	>8.3	<2.51	<2.2	<2.95
12	<23.3	>4.3	>5.4	>4	<8.3	<2.51	<2.2	<2.95
13	>23.3	>4.3	>5.4	>4	<8.3	<2.51	<2.2	<2.95
14	<23.3	>4.3	>5.4	<4	<8.3	<2.51	<2.2	<2.95
15	<23.3	>4.3	>5.4	<4	<8.3	<2.51	<2.2	<2.95
16	<23.3	>4.3	>5.4	>4	<8.3	<2.51	<2.2	<2.95

Abbreviations: Diameter RA, transverse dimension of the right atrium of the heart; Length RA, longitudinal dimension of the right atrium of the heart; Diameter RV, transverse dimension of the right ventricle of the heart; Length RV, longitudinal dimension of the right ventricle of the heart; Diameter PA I, diameter of the pulmonary artery behind the division into the right and left branches; Diameter PA II, diameter of the pulmonary artery at the site of donation I segmental branch; Diameter PA III, the diameter of the pulmonary artery in the middle part of the main trunk. ^a^ Pulmonary perfusion in the upper field.

**Table 10:** Comparison of pulmonary perfusion in the upper field, transverse and longitudinal dimensions of the right atrium and right ventricle, and the diameter of the pulmonary artery at individual measurement points relative to the literature standards 5–10 years after pneumonectomy.

Patient	Perfusion, ^a^ %	Diameter RA, cm	Length RA, cm	Diameter RV, cm	Length RV, cm	Diameter PA I, cm	Diameter PA II, cm	Diameter PA III, cm
1	<23.3	<4.3	>5.4	>4	<8.3	<2.51	<2.2	<2.95
2	<23.3	<4.3	<5.4	>4	>8.3	<2.51	<2.2	<2.95
3	<23.3	<4.3	<5.4	<4	<8.3	<2.51	<2.2	<2.95
4	<23.3	<4.3	>5.4	>4	>8.3	<2.51	<2.2	<2.95

Abbreviations: Diameter RA, transverse dimension of the right atrium of the heart; Length RA, longitudinal dimension of the right atrium of the heart; Diameter RV, transverse dimension of the right ventricle of the heart; Length RV, longitudinal dimension of the right ventricle of the heart; Diameter PA I, diameter of the pulmonary artery behind the division into the right and left branches; Diameter PA II, diameter of the pulmonary artery at the site of donation I segmental branch; Diameter PA III, the diameter of the pulmonary artery in the middle part of the main trunk. ^a^ Pulmonary perfusion in the upper field.

**Table 11:** Comparison of pulmonary perfusion in the upper field, transverse and longitudinal dimensions of the right atrium and right ventricle, and the diameter of the pulmonary artery at individual measurement points relative to the literature standards 10 years after pneumonectomy.

Patient	Perfusion, ^a^ %	Diameter RA, cm	Length RA, cm	Diameter RV, cm	Length RV, cm	Diameter PA I, cm	Diameter PA II, cm	Diameter PA III, cm
1	>23.3	<4.3	<5.4	>4	>8.3	>2.51	>2.2	<2.95
2	>23.3	<4.3	>5.4	>4	<8.3	<2.51	>2.2	<2.95
3	>23.3	>4.3	>5.4	>4	<8.3	<2.51	>2.2	>2.95
4	>23.3	>4.3	>5.4	>4	<8.3	>2.51	>2.2	>2.95

Abbreviations: Diameter RA, transverse dimension of the right atrium of the heart; Length RA, longitudinal dimension of the right atrium of the heart; Diameter RV, transverse dimension of the right ventricle of the heart; Length RV, longitudinal dimension of the right ventricle of the heart; Diameter PA I, diameter of the pulmonary artery behind the division into the right and left branches; Diameter PA II, diameter of the pulmonary artery at the site of donation I segmental branch; Diameter PA III, the diameter of the pulmonary artery in the middle part of the main trunk. ^a^ Pulmonary perfusion in the upper field.

## Data Availability

Data available on request due to ethical reasons. Patient’s records are kept in the archives of the Department of Thoracic Surgery and Transplantation, Pomeranian Medical University, Szczecin, Poland.

## Abbreviations

The following abbreviations are used in this manuscript:

PH	Pulmonary hypertension
RV	Right ventricle
PA	Pulmonary artery
RA	Right atrium
PPE	Postpneumonectomy empyema
TAPSE	Tricuspid Annular Plane Systolic Excursion
sPAP	Systolic pulmonary artery pressure
